# Childhood Nephrotic Syndrome Management and Outcome: A Single Center Retrospective Analysis

**DOI:** 10.1155/2017/2029583

**Published:** 2017-02-23

**Authors:** Chia-shi Wang, Jia Yan, Robert Palmer, James Bost, Mattie Feasel Wolf, Larry A. Greenbaum

**Affiliations:** ^1^Emory University School of Medicine, 2015 Uppergate Drive NE, Atlanta, GA 30322, USA; ^2^Children's Healthcare of Atlanta, 1677 Tullie Circle, Atlanta, GA 30329, USA

## Abstract

There is a paucity of information on outpatient management and risk factors for hospitalization and complications in childhood nephrotic syndrome (NS). We described the management, patient adherence, and inpatient and outpatient usage of 87 pediatric NS patients diagnosed between 2006 and 2012 in the Atlanta Metropolitan Statistical Area. Multivariable analyses were performed to examine the associations between patient characteristics and disease outcome. We found that 51% of the patients were treated with two or more immunosuppressants. Approximately half of the patients were noted to be nonadherent to medications and urine protein monitoring. The majority (71%) of patients were hospitalized at least once, with a median rate of 0.5 hospitalizations per patient year. Mean hospital length of stay was 4.0 (3.8) days. Fourteen percent of patients experienced at least one serious disease complication. Black race, frequently relapsing/steroid-dependent and steroid-resistant disease, and the first year following diagnosis were associated with higher hospitalization rates. The presence of comorbidities was associated with longer hospital length of stay and increased risk of serious disease complications. Our results highlight the high morbidity and burden of NS and point to particular patient subgroups that may be at increased risk for poor outcome.

## 1. Introduction

Idiopathic nephrotic syndrome (NS) is one of the most common chronic kidney diseases in children, with a prevalence of approximately 16 cases per 100,000 [1]. It is characterized by heavy proteinuria, hypoalbuminemia, edema, and hyperlipidemia. During active disease, the loss of proteins critical for various biologic functions can result in complications such as infections, thromboembolic disease, and acute kidney injury [1–4]. The current mainstay of treatment is high-dose oral corticosteroids. However, 80 to 90% percent of patients will experience disease relapse, with half relapsing frequently or becoming dependent on corticosteroids to maintain remission. In addition, approximately 7.4–19.6% of children have corticosteroids-resistant disease with poor renal prognosis [5–7]. Repeated and prolonged use of corticosteroids can have adverse effects on metabolism, growth, and behavior [8]. Second-line immunosuppressive agents, such as calcineurin inhibitors, cytotoxic agents, mycophenolate mofetil, and rituximab, are given to those intolerant or resistant to corticosteroids. These agents can cause additional side effects and have expected response rates of only 20–50% [9].

Research has underscored the morbidity and burden of childhood NS. Focal segmental glomerulosclerosis (FSGS), one of the most common histologic variants of NS, is the second-most common cause of end-stage renal disease (ESRD) in the North American Pediatric Renal Transplant Cooperative Study [10]. A cross-sectional analysis of the Kids' Inpatient Database from the Healthcare Cost and Utilization Project (HCUP-KID) revealed that NS resulted in an estimated 48,700 inpatient days and charges totaling $259 million nationally in the years 2006 and 2009. Furthermore, 16% of the discharges had at least one severe complication, including thromboembolism, septicemia, peritonitis, pneumonia, or diabetes [11].

There is a great need to examine the influences on disease outcome in childhood NS. Management of childhood NS involves intense outpatient follow-up and family participation for disease monitoring and treatment. Despite the complexity, few reports exist that describe management patterns. Survey studies have noted significant differences in provider preference for second-line immunosuppressants, glucocorticoid regimens, and when renal biopsies are performed [12, 13]. There are no reports on outpatient clinic visit usage or patient adherence to urine monitoring and medications. There are limited studies on risk factors for increased inpatient usage among pediatric NS patients. Gipson et al. identified patient age 15 years or older, black race, higher socioeconomic status, acute renal failure, thromboembolic disease, hypertension, and infections to be associated with higher inpatient charges [11]. No information is available on predicators for hospitalizations.

We thus performed a retrospective study to assess the relationships between patient characteristics and disease outcome. A thorough review of inpatient and outpatient charts for NS patients was conducted at Children's Healthcare of Atlanta (CHOA), the sole provider of pediatric nephrology care in the Atlanta Metropolitan Statistical Area (MSA). We were able to capture every inpatient and outpatient nephrology encounter and provide descriptions of outpatient management and inpatient usage, which had not previously been reported in pediatric NS. Our goals were to provide an in-depth look at outpatient disease management and to determine risk factors for inpatient utilization and disease complications.

## 2. Materials and Methods

### 2.1. Setting

A retrospective chart review was performed on pediatric NS patients diagnosed and managed by the Division of Pediatric Nephrology at Emory University. Outpatient and inpatient charts for up to 3 years from the time of diagnosis were reviewed. The Division of Pediatric Nephrology at Emory University includes all the pediatric nephrologists practicing in the Atlanta MSA and provides all of the nephrology care at the three campuses of CHOA, the only pediatric hospitals within the Atlanta MSA. We were thus able to capture all renal outpatient encounters and all inpatient encounters. The study was approved by the CHOA institutional review board.

### 2.2. Study Population

We screened patients followed by the Division of Pediatric Nephrology at Emory University for the diagnosis of NS by using* International Classifications of Diseases, Ninth Revision, Clinical Modification* (ICD-9 CM) diagnostic codes 581.3, 581.9, and 582.1. A single pediatric nephrologist (Chia-shi Wang, MD) reviewed each patient's demographic, clinical, laboratory, and histologic information for inclusion and exclusion. We included patients who were >1 and <18 years of age at the onset of NS, met the clinical diagnosis of idiopathic NS (edema, nephrotic range proteinuria, and hypoalbuminemia), resided in the Atlanta MSA at time of diagnosis and during the entire period of follow-up, and were diagnosed between 1/1/2006 and 1/1/2012. Patients who had been hospitalized outside of CHOA during the follow-up period were excluded. Other exclusion criteria included renal biopsy findings other than minimal change disease (MCD), FSGS, or a variant (mesangial proliferation, IgM deposits, C1q deposits); ESRD; renal transplantation; or secondary causes of nephrotic syndrome (e.g., systemic lupus erythematosus).

### 2.3. Measurements

Clinical, demographic, and outpatient care adherence characteristics were collected from outpatient records. Variables of interest included sex, age at time of diagnosis, race, ethnicity, insurance type at time of diagnosis, renal histopathology (if biopsy performed), presence of comorbid disease at time of diagnosis (epilepsy, congenital heart disease, inflammatory bowel disease, chronic lung disease, asthma/airway reactive disease, prematurity, or other), NS disease status by corticosteroid response, and use of second-line immunosuppressive agents (e.g., mycophenolate, tacrolimus). NS disease status is classified based on the definitions in Kidney Disease, Improving Global Outcomes [8]. Patients are classified as having steroid-resistant NS (SRNS) if they fail to achieve remission after eight weeks of corticosteroid therapy. Among patients who achieve remission within eight weeks of starting corticosteroid therapy, the disease is classified as frequently relapsing NS (FRNS) if there are two or more relapses within six months of initial response or four or more relapses in any 12-month period; steroid-dependent NS (SDNS) if there are two consecutive relapses during corticosteroid taper, or within 14 days of ceasing therapy; otherwise, the disease is classified as steroid-sensitive, infrequently relapsing NS (SSNS). The classification is made based on the clinical response to corticosteroids in the first three years following diagnosis.

All outpatient nephrology clinic visits pertaining to the treatment and monitoring of NS were reviewed, including both routinely scheduled and acute visits. The numbers of completed and missed clinic appointments were recorded. Percent “no-show” was the number of missed appointments divided by the total number of clinic appointments. A patient was considered as nonadherent with prescribed clinic visits if they had >20% “no-show” during the follow-up period. Assessment of adherence to home urine protein monitoring and medications were based on the documentation of treating physicians who subjectively noted good or poor adherence.

Inpatient records for up to three years from time of diagnosis were reviewed. Each hospitalization was reviewed for its relationship to NS. Only hospitalizations indicated for the management of NS complications or treatment side-effects were included. The number of hospitalizations, length of stay (LOS), intensive care unit (ICU) status, and serious complications were recorded. A serious complication was defined as one of the following: bacterial peritonitis (with or without culture confirmation), septicemia, shock, blood clot(s) (radiologically confirmed), acute kidney injury requiring dialysis, or seizures from hyponatremia or hypertension. Edema, asymptomatic electrolyte abnormalities, and asymptomatic hypertension were not considered serious complications. Disease complications were recorded for each patient if they were the reasons for hospitalization or if they occurred during hospitalization.

### 2.4. Statistical Analysis

Demographic and clinical characteristics were described by number of patients and percentages. The number of occurrences and percentage of patients were computed for hospitalizations and serious disease complications. Mean and standard deviation or median and interquartile range, where appropriate, were computed for number of outpatient clinic visits, number of clinic appointment “no-shows,” hospitalizations, and length of stay (LOS).

Associations of clinical and demographic factors with the number of hospitalizations per year were assessed with Poisson regression. We applied generalized estimating equations to the Poisson distribution to account for within-patient correlation. Independent variables were considered for the model based on clinical relevance and include age, sex, race and ethnicity, insurance status, disease status, comorbidities, and year since diagnosis. Renal histopathology was not included in the model as a large number of patients did not undergo biopsy. Adherence and use of second-line immunosuppressants variables were not included in the model due to difficulty delineating the causal pathway, that is, whether adherence/medications influences the risk of hospitalizations or whether hospitalizations influence the likelihood of adherence to therapy/medications prescribed. Parameter estimates from the model were exponentiated to produce rate ratios.

Associations of clinical and demographic factors with the development of severe disease complications in patients over the duration of follow-up were assessed with logistic regression. Independent variables included in the model were determined a priori based on clinical significance and included age, sex, race and ethnicity, insurance, disease status, and comorbidities. Model fit was assessed by the Hosmer-Lemeshow test.

Linearity of the continuous outcome variable LOS in days per hospitalization was assessed graphically and by skewness and kurtosis measures. Logarithmic transformation of LOS was carried out due to high skewness. Associations between the clinical and demographic factors with LOS were assessed with linear regression. Parameter estimates were then back-transformed to the linear scale to produce mean proportional change in LOS.

Significance level for tests of association was set at 0.05. Statistical analysis was performed using the SAS system, version 9.4 (SAS Institute, Cary, NC).

## 3. Results

### 3.1. Patient Characteristics

A total of 87 patients contributed data. All had complete inpatient and outpatient records for 1 year following diagnosis. Eight-two patients (94%) had complete records for 2 years, and 80 patients (92%) had complete records for 3 years. Reasons for lacking year 2 and year 3 records were discharge from clinic (2 out of 87, 2%) and lost to follow-up (5 out of 87, 6%). Clinical and demographic characteristics are shown in Table 1. The majority of patients had steroid sensitive disease (81.6%), with 55% dependent on corticosteroids and relapsing frequently. Eighteen percent of the patients had SRNS. The proportion of patients in each disease category was comparable to prior findings [9, 14]. Patient with SRNS tended to be older and had a more equal male to female ratio compared to those with SSNS and FRNS/SDNS. The majority of SRNS patients were also black, in contrast to patients with SSNS and FRNS/SDNS. The demographic breakdown of our patients was also similar to prior reports [1]. A greater proportion of SRNS patients underwent renal biopsy.

### 3.2. Outpatient Management

The management and adherence patterns of patients by NS classification are shown in Table 2. Patients had an overall mean (SD) of 3.7 (2.0) clinic visits per year, with a mean of 5.0 (2.0) visits in the first year, 3.0 (1.6) visits in the second year, and 2.8 (1.6) visits in the third year. On average, patients with SSNS had the least number of prescribed clinic visits (2.59, 1.1), followed by patients with FRNS/SDNS (4.33, 1.7). On average, patients with SRNS had the most number of prescribed clinic visits (4.71, 1.7). All patients were initially treated with corticosteroids for a minimum of 6 weeks on presentation. Forty-four patients (51%) were subsequently treated with at least one additional immunosuppressive agent. These 44 patients had either FRNS/SDNS or SRNS disease. The choice of agents differed between FRNS/SDNS and SRNS patients. The preferred agent for patients with FRNS/SDNS was mycophenolate mofetil, selected first in 21 of the 29 (72%) patients who received agents in addition to corticosteroids. In contrast, the preferred agent for patients with SRNS was cyclosporine, selected first in seven out of 12 (58%) patient who received agents in addition to corticosteroids.

Thirty-two out of 71 patients (45%) who were prescribed urine dipsticks for home monitoring were subjectively noted by their treating physicians to be nonadherent with urine monitoring. Thirty-seven out of 87 (43%) patients were noted to be poorly adherent to prescribed NS medications. Twenty-eight out of 87 (32%) of patients had >20% “no-shows” to clinic appointments. Mean percentage of “no-shows” was 14% (22), with a mean of 11% (16) in the first year, 15% (24) in the second year, and 19% (25) in the third year.

### 3.3. Inpatient Care Utilization

A total of 184 hospitalizations were recorded for the 87 patients. Median hospitalization rate was 0.5 hospitalization per patient year (interquartile range = 0.0 to 1.0). Sixty-two patients (71%) had at least one hospitalization, and 13 patients (15%) had 5 or more hospitalizations in the first 3 years following diagnosis. The mean (SD) LOS per hospitalization was 4.0 (3.8) days.

### 3.4. Complications

Four out of 184 hospitalizations (2%) resulted in an ICU stay. Reasons for the ICU stays were respiratory distress due to pleural effusion in a child with FRNS/SDNS, respiratory distress due to bacterial pneumonia in a child with FRNS/SDNS, hyponatremia in a child with FRNS/SDNS, and seizures from hypertension in a child with SRNS. Twelve of the 87 (14%) patients experienced serious complications in the first 3 years of disease: two patients with SSNS, seven patients with FRNS/SDNS, and 3 patients with SRNS. Eight patients (9%) experienced bacterial peritonitis, 4 patients (4.6%) experienced septicemia or shock, 2 (2%) experienced blood clots, 1 patient (1%) required renal replacement therapy for acute kidney injury, and 1 patient (1%) developed seizures from hypertension.

### 3.5. Predictors for NS Morbidity

Our multivariable analysis showed that FRNS/SDNS and SRNS were associated with >4-times higher rates of hospitalization (rate ratio (RR) = 4.43, 95% confidence interval (CI) = 2.74 to 7.15; and RR = 4.14, CI = 1.64 to 10.44; resp.), relative to SSNS. Black race was also significantly associated with increased hospitalization rates (RR = 1.84, CI = 1.04 to 3.25), compared to white race. The hospitalization rates were lower in the second and third year of disease, compared to the first year (RR = 0.35, CI = 0.25 to 0.51; and RR = 0.26, CI = 0.16 to 0.41; resp.). Age at diagnosis, sex, insurance status, and presence of comorbidities were not significantly associated with the hospitalization rate in this analysis. The results of the analysis are presented in Table 3.

Multivariable analysis of LOS results is presented in Table 4. The presence of comorbidities and severe disease complications were associated with higher LOS (mean increase of 42% (CI = 5% to 93%) and 43% (CI = 6% to 92%), resp.). On average, hospital LOS for black patients was 28% shorter than for white patients (CI = 0.52 to 1.00). Age at diagnosis, sex, insurance status, and disease status were not significantly associated with LOS in this analysis.

Only the presence of comorbidities was found to be associated with the risk of serious complications (OR = 5.36, CI = 1.26 to 22.87). Age at diagnosis, sex, race and ethnicity, insurance status, and disease status were not found to be significantly associated with serious complications. The results of the regression analysis are presented in Table 5.

## 4. Discussion

Our findings support previous published reports that childhood NS is a disease of high morbidity and results in significant healthcare burden. The median hospitalization rate of our cohort was high at 0.50 hospitalization per patient year, and the majority of patients were hospitalized at least once (71%). On average, hospitalizations lasted approximately 4 days. Serious complications occurred in 14% of the patients in the first 3 years of follow-up. These results suggest that each NS patient has the potential to contribute significant burden to the healthcare system.

Outpatient management of our patient cohort was also resource-intensive. Clinic visits averaged 3.7 per year in the first 3 years of diagnosis. More than half of the patients were treated with second-line immunosuppressants in addition to corticosteroids, carrying additional monitoring needs. Significantly, providers noted nonadherence to urine monitoring and medications in nearly half of the patients. Furthermore, the rates of “no-shows” to appointments were high at an average of 14%. This is a serious concern as nonadherence is a major cause of treatment failure in pediatric chronic diseases [15].

We hypothesized that disease classification based on steroid response and frequency of relapse would be a predictor of increased hospitalizations and complications, as it is an important determinant of disease prognosis [1]. This was substantiated in our analysis on hospitalizations. FRNS/SDNS and SRNS were associated with increased hospitalization rates compared to children with SSNS. Patients were hospitalized more frequently in the first year of diagnosis. Black race was also found to be associated with increased hospitalization rates despite controlling for disease status and clinical and demographic factors. This may mean that there are other social, economic, or provider/patient factors not captured by our analysis. Disease classification was not associated with increased risk of serious complications as we had hypothesized. It may be possible that our sample size and the number of serious complications were too small to detect a significant difference. Similar to analyses of the HCUP-KID data [11, 16], serious NS complications were associated with longer hospitalizations. In our analysis, black patients had shorter hospitalizations compared to white patients after controlling for disease status, complications, and other clinical and demographic factors. This again suggests the need to explore determinants of health and disease management that result in racial differences. The presence of any comorbid condition, not surprisingly, was significantly associated with increased LOS and development of serious complications. Our analyses suggest that patients with FRNS/SDNS or SRNS with other comorbid conditions may be a particularly vulnerable group.

Our study provided a detailed description of inpatient and outpatient care usage in children with NS. The strengths of our single center analysis include the ability to accurately define a cohort of incident patients with idiopathic NS and capture all instances of disease complications without relying on ICD-9 CM diagnostic codes. We were able to report for the first time hospitalization rates, outpatient clinic visit rates, frequency of various immunosuppressant usage, and adherence in children with NS. We were also able to study the influence of NS disease classification on outcomes.

As a single center report, our findings on inpatient and outpatient usage may have limited generalizability. Due to the restrictive inclusion and exclusion criteria with respect to patient address, our sample size is small. This may have affected the validity of our multivariable analyses and reduced our ability to detect significant associations. In addition, as a retrospective study, we were unable to obtain objective measures for medication and urine monitoring adherence or delineate the causal pathway between adherence and NS disease outcome, a variable we suspect to be crucial in chronic disease outcome. Lastly, the duration of follow-up for our cohort is relatively short. Thus, we did not assess rates of important complications such as end-stage renal disease.

## 5. Conclusions

In conclusion, our study examined NS disease management and inpatient and outpatient usage and described the associations between clinical and demographic characteristics and disease outcome. Our results suggest that patients with FRNS/SDNS and SRNS with other comorbidities are a vulnerable group. Multicenter, prospective studies would enhance our understanding of how outpatient management and adherence patterns of NS patients influence outcome.

## Figures and Tables

**Table 1:** Characteristics of patients with childhood nephrotic syndrome diagnosed between 2006 and 2012 in the Atlanta MSA.

Characteristic	SSNS (*n* = 23)	FRNS/SDNS (*n* = 48)	SRNS (*n* = 16)	Total (*n* = 87)
*Sex*				
Male	17 (74)	32 (67)	9 (56)	58 (67)
Female	6 (26)	16 (33)	7 (44)	29 (33)
*Age at diagnosis*				
1–5 years	17 (74)	27 (56)	3 (19)	47 (54)
6–12 years	6 (26)	15 (31)	5 (31)	26 (30)
13–18 years	0 (0)	6 (13)	8 (50)	14 (16)
*Race and ethnicity*				
White, non-Hispanic	6 (26)	15 (31)	2 (13)	23 (26)
Black	7 (30)	19 (40)	13 (81)	39 (45)
White, Hispanic	4 (17)	9 (19)	1 (6)	14 (16)
Other	6 (26)	5 (10)	0 (0)	11 (13)
*Insurance*				
Private	11 (48)	22 (46)	6 (38)	39 (45)
Medicaid	11 (48)	23 (48)	8 (50)	42 (48)
None	1 (4)	3 (6)	2 (13)	6 (7)
*Histopathology*				
No biopsy	22 (96)	27 (56)	2 (13)	51 (59)
MCD	0 (0)	17 (35)	5 (31)	22 (25)
FSGS	1 (4)	1 (2)	6 (38)	8 (9)
Other	0 (0)	3 (6)	3 (19)	6 (8)
*Comorbidity*				
None	19 (83)	40 (83)	11 (69)	70 (80)
Asthma	3 (13)	7 (15)	1 (6)	11 (13)
Epilepsy	1 (4)	0 (0)	2 (13)	3 (3)
Inflammatory bowel disease	0 (0)	1 (2)	1 (6)	2 (2)
Prematurity	0 (0)	2 (4)	0 (0)	2 (2)
Other	1 (4)	1 (2)	1 (6)	3 (3)

Data are presented as the number of patients with the percentage in parenthesis.

FRNS, frequently relapsing nephrotic syndrome; MCD, minimal change disease; FSGS, focal segmental glomerulosclerosis; SDNS, steroid-dependent nephrotic syndrome; SRNS, steroid-resistant nephrotic syndrome; SSNS, steroid-sensitive and infrequently relapsing nephrotic syndrome.

**Table 2:** Outpatient management and adherence patterns by nephrotic syndrome classification.

Management	SSNS (*n* = 23)	FRNS/SDNS (*n* = 48)	SRNS (*n* = 16)	Total (*n* = 87)
Mean clinic visits per year (SD)	2.59 (1.1)	4.33 (1.7)	4.71 (1.7)	3.7 (2.0)
*Immunosuppressive treatment*				
Corticosteroids only	23 (100)	19 (40)	4 (25)	46 (53)
Mycophenolate mofetil	0 (0)	24 (50)	6 (38)	30 (34)
Cyclophosphamide	0 (0)	7 (15)	2 (13)	9 (10)
Tacrolimus	0 (0)	7 (15)	3 (19)	10 (11)
Cyclosporine	0 (0)	2 (4)	8 (50)	10 (11)
>1 agents in addition to corticosteroids	0 (0)	10 (21)	4 (25)	14 (16)
*Medication adherence*				
Adherent	18 (78)	24 (50)	8 (50)	50 (57)
Nonadherent	5 (22)	24 (50)	8 (50)	37 (43)
*Clinic follow-up adherence*				
Adherent	15 (65)	35 (73)	9 (56)	60 (70)
Nonadherent	8 (35)	13 (27)	7 (44)	28 (32)

				Total (*n* = 71)

*Urine monitoring*				
Adherent	15 (65)	24 (50)	N/A	39 (55)
Nonadherent	8 (35)	24 (50)	N/A	32 (45)

Data are presented as the number of patients with the percentage in parenthesis or mean and standard deviation in parenthesis.

FRNS, frequently relapsing nephrotic syndrome; MCD, minimal change disease; FSGS, focal segmental glomerulosclerosis; SD, standard deviation; SDNS, steroid-dependent nephrotic syndrome; SRNS, steroid-resistant nephrotic syndrome; SSNS, steroid-sensitive and infrequently relapsing nephrotic syndrome.

**Table 3:** Association between patient characteristics and hospitalization rate.

Variables	Adjusted RR (95% CI)	*P* value
*Age*		
1–5 years	1.00 (referent)	
6–12 years	1.10 (0.63 to 1.91)	0.74
13–18 years	1.11 (0.52 to 2.36)	0.79
*Sex*		
Male	1.00 (referent)	
Female	0.82 (0.55 to 1.23)	0.34
*Race and ethnicity*		
White, non-Hispanic	1.00 (referent)	
Black	1.84 (1.04 to 3.25)	0.04^*∗*^
White, Hispanic	1.41 (0.65 to 3.07)	0.38
Other	1.11 (0.56 to 2.21)	0.76
*Insurance*		
None	1.0 (referent)	
Medicaid	1.15 (0.59 to 2.23)	0.67
Private	0.74 (0.36 to 1.57)	0.45
*Disease status*		
SSNS	1.00 (referent)	
FRNS/SDNS	4.43 (2.74 to 7.15)	<0.001^*∗*^
SRNS	4.14 (1.64 to 10.44)	0.003^*∗*^
*Comorbidity*		
No	1.00 (referent)	
Yes	1.33 (0.75 to 2.37)	0.33
*Year since diagnosis*		
1	1.00 (referent)	
2	0.35 (0.24 to 0.51)	<0.001^*∗*^
3	0.26 (0.16 to 0.41)	<0.001^*∗*^

CI, confidence interval; FRNS, frequently relapsing nephrotic syndrome; RR, rate ratio; SDNS, steroid-dependent nephrotic syndrome; SRNS, steroid-resistant nephrotic syndrome; SSNS, steroid-sensitive and infrequently relapsing nephrotic syndrome.

^*∗*^ *P* < 0.05.

**Table 4:** Association between patient characteristics and length of stay among hospitalized patients.

Variables	Adjusted *β* estimate^a^ (95% CI)	*P* value
*Age*		
1–5 years	1.00 (referent)	
6–12 years	1.21 (0.89 to 1.65)	0.21
13–18 years	1.24 (0.88 to 1.75)	0.21
*Sex*		
Male	1.00 (referent)	
Female	1.11 (0.86 to 1.43)	0.41
*Race and ethnicity*		
White, non-Hispanic	1.00 (referent)	
Black	0.72 (0.52 to 1.00)	0.05^*∗*^
White, Hispanic	0.76 (0.51 to 1.14)	0.18
Other	0.61 (0.37 to 1.01)	0.06
*Insurance*		
None	1.00 (referent)	
Medicaid	0.83 (0.53 to 1.30)	0.41
Commercial	0.74 (0.46 to 1.19)	0.21
*Disease status*		
SSNS	1.00 (referent)	
FRNS/SDNS	0.81 (0.59 to 1.12)	0.20
SRNS	0.87 (0.58 to 1.29)	0.47
*Comorbidity*		
No	1.00 (referent)	
Yes	1.42 (1.05 to 1.93)	0.03^*∗*^
*Complications*		
No	1.00 (referent)	
Yes	1.43 (1.06 to 1.92)	0.02^*∗*^

CI, confidence interval; FRNS, frequently relapsing nephrotic syndrome; SDNS, steroid-dependent nephrotic syndrome; SRNS, steroid-resistant nephrotic syndrome; SSNS, steroid-sensitive and infrequently relapsing nephrotic syndrome.

^a^After exponentiation to give proportional change in mean length of stay (days) compared to the referent.

^*∗*^ *P* < 0.05.

**Table 5:** Association between patient characteristics and serious complications.

Variables	Adjusted OR (95% CI)	*P* value
*Age*		
1–5 years	1.00 (referent)	
6–12 years	0.64 (0.11 to 3.75)	0.62
13–18 years	1.42 (0.19 to 10.65)	0.74
*Sex*		
Male	1.00 (referent)	
Female	0.83 (0.18 to 3.86)	0.81
*Race and ethnicity*		
White, non-Hispanic	1.00 (referent)	
Black	1.90 (0.30 to 12.19)	0.50
White, Hispanic	0.97 (0.06 to 14.89)	0.98
Other	1.36 (0.09 to 21.12)	0.83
*Insurance*		
None	1.00 (referent)	
Medicaid	0.31 (0.03 to 2.99)	0.31
Commercial	0.29 (0.03 to 3.01)	0.30
*Disease status*		
SSNS	1.00 (referent)	
FRNS/SDNS	1.81 (0.30 to 10.87)	0.52
SRNS	1.17 (0.10 to 13.89)	0.90
*Comorbidity*		
No	1.00 (referent)	
Yes	5.36 (1.26 to 22.87)	0.02^*∗*^

CI, confidence interval; FRNS, frequently relapsing nephrotic syndrome; SDNS, steroid-dependent nephrotic syndrome; SRNS, steroid-resistant nephrotic syndrome; SSNS, steroid-sensitive and infrequently relapsing nephrotic syndrome.

^*∗*^ *P* < 0.05.
